# Advancements in fire-related toxic gas detection and prophylactic strategies: A focus on cyanide concentration analysis and antidote efficacy in controlled smoke inhalation models

**DOI:** 10.1371/journal.pone.0333779

**Published:** 2026-06-15

**Authors:** Jowy Tani, Jia-Long Chen, Wei-Chuan Liao, Chia-Hung Chen, Chau-Hui Wang, Tzu-hui Sun, Yow-Ling Shiue, Jin-Wu Tsai

**Affiliations:** 1 Department of Neurology, School of Medicine, College of Medicine, Taipei Medical University‌‌, Taipei, Taiwan; 2 Department of Neurology, Wan Fang Hospital, Taipei Medical University, Taipei‌‌, Taiwan; 3 Original Biomedicals Co., Ltd., Southern Taiwan Science Park, Tainan, Taiwan; 4 Institute of Biomedical Sciences, National Sun Yat-sen University‌‌, Kaohsiung, Taiwan; 5 Institute of Precision Medicine, National Sun Yat-sen University, Kaohsiung, Taiwan; 6 Institute of Brain Science, School of Medicine, National Yang-Ming Chiao Tung University, Taipei, Taiwan; Hanyang University - Seoul Campus: Hanyang University, KOREA, REPUBLIC OF

## Abstract

Fire smoke inhalation represents a major cause of acute mortality in fire incidents, with hydrogen cyanide being a critical contributor to rapid systemic toxicity. This study aimed to establish a controlled smoke inhalation model to characterize cyanide-dominant exposure and to evaluate the prophylactic efficacy of a nebulized antidote combination under acute conditions. A reproducible smoke chamber system was developed to generate cyanide-rich toxic atmospheres. C57BL/6 mice were exposed to controlled cyanide concentrations, followed by prophylactic administration of a nebulized formulation containing hydroxocobalamin and deferoxamine. Survival outcomes were assessed to evaluate the protective effects of the intervention. Controlled cyanide exposure resulted in rapid and dose-dependent lethality. Prophylactic inhalation of the nebulized antidote significantly improved short-term survival compared with untreated controls (*p* < 0.05), consistent with effective mitigation of cyanide toxicity. In conclusion, this study establishes a reproducible cyanide-focused smoke inhalation model and provides experimental evidence supporting the potential of aerosolized hydroxocobalamin-based prophylaxis as an immediate protective strategy during fire smoke exposure. These findings support the feasibility of rapid, non-invasive intervention aimed at preserving a critical time window for escape and subsequent medical treatment in fire-related emergencies.

## Introduction

In addition to rising global temperatures and increasing extreme weather events, the use of modern electrical equipment has become a significant factor in fire incidents. Beyond acute fire incidents, increasing wildfire smoke exposure has also emerged as a significant public health concern, disproportionately affecting vulnerable populations and raising issues of environmental justice [1]. Climate-driven increases in ambient temperature have been shown to significantly elevate the frequency and intensity of wildfire events, thereby increasing population-level exposure to toxic fire smoke [2]. For example, the proliferation of electric vehicle charging facilities and overloaded circuits have substantially increased the risk of electrical fires. The International Association of Fire and Rescue Services (CTIF) reports that electrical fires account for a notable proportion of global fire incidents, with firefighter fatalities associated with fire events also on the rise [3]. Smoke inhalation remains a major cause of morbidity and mortality in fire incidents, and toxic gases generated during combustion are key contributors to early fatalities.

In addition to wildfires, industrial fires are another major source of toxic fire smoke. The combustion of various chemicals used in industrial facilities releases highly toxic gases, posing severe health risks. Cyanide, in particular, is a dangerous component of fire smoke, produced during the combustion of nitrogen-containing materials such as melamine, wool, and silk. Cyanide rapidly inhibits cellular respiration, leading to hypoxia and, if untreated, can be fatal [4].

Smoke inhalation is a significant cause of morbidity and mortality in fire incidents. The gases, particulates, and toxic substances, such as cyanide, present in smoke pose severe threats to fire victims and firefighters. Cyanide is a potent cellular toxin whose rapid inhibition of cellular respiration makes it a critical component of fire smoke toxicity. Particularly in industrial fires, the combustion of melamine can produce extremely high and lethal concentrations of cyanide. At high temperatures, melamine combustion generates cyanide in quantities sufficient to be immediately fatal, posing extreme risks to firefighters entering fire environments [5]. These conditions pose particular dangers in enclosed or industrial fire environments where nitrogen-containing materials are abundant.

Given the multifaceted issue of fire smoke toxicity, this study investigated cyanide concentrations and their acute toxic effects in smoke. By conducting controlled experiments in a smoke chamber with cyanide concentrations set at 100–110 ppm, we quantified the lethal concentration of cyanide in fire smoke and examined its dynamics in a controlled environment [4,5].

Cyanide is frequently generated through the combustion of various synthetic materials, including foams like melamine foam. Due to cyanide’s rapid inhibition of cellular respiration, it can cause severe toxic reactions. This study avoids the erroneous analogy between “cyanide exposure and vitamin B12 deficiency” and instead focuses on cyanide toxicity and the established mechanisms of vitamin B12, particularly hydroxocobalamin, in detoxification [6].

Accordingly, cyanide exposure was evaluated independently as an acute toxic insult rather than as a nutritional deficiency model.

Beyond assessing cyanide toxicity in smoke, this study further evaluated the efficacy of rescue drugs, such as hydroxocobalamin (Cyanokit), in mitigating cyanide’s toxic effects [7]. Existing literature indicates that hydroxocobalamin is an effective antidote for cyanide poisoning in smoke inhalation scenarios. This work contributes to this body of knowledge by comparing the survival rates of treated and untreated subjects in controlled experiments [8–10].

Mice were used as experimental models due to their physiological similarities to humans in toxicology studies. Survival rates and survival times were recorded in the smoke chamber to provide valuable data on cyanide’s lethality and antidote effectiveness [5,6].

Considering the complexity and potential variability of fire smoke composition, validated analytical methods were employed, including Ultra Performance Liquid Chromatography-Mass Spectrometry (UPLC-MS) for drug concentration analysis, while cyanide concentrations were quantified using colorimetric methods [11,12].

The ultimate goal of this study was to provide insights that can inform the development of more effective fire smoke prevention strategies and treatment protocols. By understanding the specific risks associated with cyanide in fire smoke and evaluating the efficacy of existing antidotes, this work contributes to a body of knowledge that helps protect civilians and firefighters from the dangers of smoke inhalation.

Additionally, our research plans to explore the potential of aerosolized antidote delivery in fire rescue operations. Recent studies have demonstrated that aerosolized hydroxocobalamin can effectively detoxify cyanide through rapid pulmonary absorption, supporting the feasibility of inhalation-based antidote delivery for acute cyanide exposure scenarios [10]. Aerosolized delivery can rapidly administer the drug to the lungs, enhancing absorption efficiency and providing immediate detoxification effects at the fire scene. This delivery method offers significant advantages for rescue personnel operating in high-risk environments [13–15].

These efforts will provide scientific foundations and practical guidance for improving fire smoke prevention and treatment, ultimately enhancing public safety and rescue efficiency.

## Materials and methods

### Animal model and ethical approval‌‌

In our study, we adhere to the highest standards of ethical treatment for the animal subjects used. Male B6(C57BL/6) mice aged 8–10 weeks were obtained from LASCO and selected for their physiological relevance and widespread use in acute toxicology studies. Each mouse is given a minimum of one week to acclimatize to the laboratory environment prior to the commencement of experiments, ensuring minimal stress and adaptation issues [16].

Humane endpoints were established in accordance with PLOS ONE and IACUC guidelines. Mice were continuously monitored during exposure and throughout the observation period. Animals exhibiting signs of severe distress, including rapid or labored breathing, marked reduction in activity, loss of righting reflex, or unconsciousness, were immediately removed from the experiment and euthanized.

Euthanasia was performed under general anesthesia induced by isoflurane inhalation to minimize pain and distress. Isoflurane anesthesia was also administered prior to terminal blood collection and other invasive procedures. Analgesia and refinement measures were implemented to minimize suffering, including close monitoring, acclimatization prior to experiments, and immediate medical intervention if unexpected adverse events occurred.

Post-experiment, all animals are continuously monitored for an additional week to observe any adverse reactions or long-term effects stemming from the experimental procedures. This extended observation period is crucial for identifying any delayed responses to the treatment or exposure, ensuring the welfare and health of the animals throughout the study duration.

Our experimental protocols strictly adhere to the principles of the 3Rs (Replacement, Reduction, and Refinement) in animal research. Approval was obtained from the Institutional Animal Care and Use Committee (IACUC) of National Yang Ming Chiao Tung University, with certification number IACUC1121001.

### Drug nebulization and safety assessment

The combination of these two drugs offers a comprehensive approach to preventing the harmful effects of fire smoke, particularly focusing on cyanide poisoning and free radical damage. The primary target of the nebulized drug combination is the respiratory tract, including the mouth, nose, bronchi, lungs, and deep alveoli. The drugs, when inhaled, provide protection along the entire respiratory pathway. They adhere to the surface of the tissues, ensuring that when toxic gases are inhaled, most of the cyanide is neutralized before entering the bloodstream. This significantly reduces the cyanide’s potential to damage blood cells and impair their oxygen-carrying function [14].

Although the concentration of inhaled drugs in the bloodstream is often low and difficult to detect, a dual-sampling strategy was used to analyze drug concentrations by collecting both blood and respiratory tissue fluid samples. UPLC-MS was employed, which can detect smaller units, to measure drug concentrations accurately [12].

The nebulizer used was HEALTH & LIFE CO., LTD.’s HL-100 model (FDA approved nebulizer, K081738). The drug combination included Hydroxocobalamin (from Tokyo Chemical Industry Co., Ltd.) and Deferoxamine (from Novartis AG), dissolved in a 0.3M Tris-buffer solution (Tris(hydroxymethyl)aminomethane). Post-inhalation, animals were monitored for one week to ensure no adverse allergic reactions or abnormalities were observed (S1 Fig in S1 File).

### Fire smoke experiment and cyanide concentration testing

To simulate a fire smoke environment with high hydrogen cyanide (HCN) concentrations, 0.02–0.03 g of melamine-based foam was combusted in a sealed 500 mL glass bottle. Upon completion of combustion, the bottle was immediately sealed to retain the generated smoke. In preliminary experiments, several combustible materials, including wood, polyurethane foam, and synthetic polymers, were evaluated for their ability to generate hydrogen cyanide (HCN). Melamine-based foam was selected due to its ability to reliably produce rapid HCN concentrations under controlled combustion conditions. This consistency was confirmed through repeated trials under standardized parameters, including material mass, chamber volume, and airflow. A conduit was then employed to directly connect the combustion bottle’s opening to the BW Ultra (Honeywell BW) HCN detector for real-time measurement of HCN concentration in ppm. Additionally, to precisely quantify HCN concentration, an aqueous sampling method was simultaneously employed. In this method, the combustion bottle was pre-filled with purified water, and after combustion, the bottle was vigorously shaken to rapidly dissolve HCN gas into the water. The obtained water samples were analyzed using an HCN detection reagent (LU HENG) via colorimetric assay, which relies on the distinct color gradient produced by varying HCN concentrations (S5 Fig in S1 File). Concentrations were then determined quantitatively by comparison to a previously established calibration curve for HCN. Based on a fixed gas flow rate and sampling time, the distribution range of the HCN concentration was thus accurately established, ensuring each experiment was conducted within the desired concentration range. Given the high toxicity of HCN, strict safety protocols were observed throughout the experiment by using appropriate personal protective equipment (e.g., gas masks, gloves), conducting all smoke tests within a fume hood, and notifying the environmental safety center in advance. After multiple trials, a stable HCN environment was established, providing a reliable basis for the fire smoke exposure model [17].

**Fig 1:**
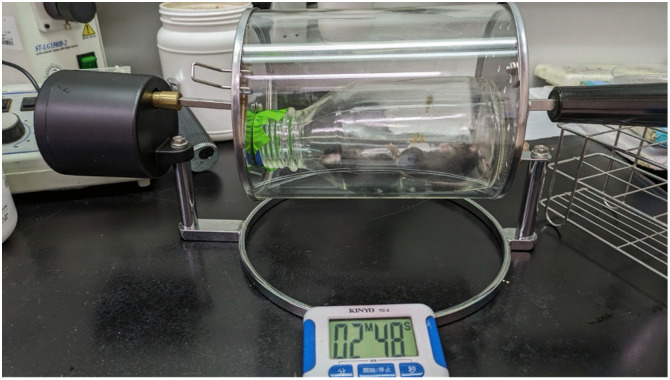
Fire smoke model.

Mice were divided into two groups for the fire smoke efficacy test. One group inhaled experimental water as a control, while the other inhaled the drug combination as a preventative measure. In a small-scale fire model, one mouse from each group (control and drug) was exposed to cyanide concentrations controlled between 100–110 ppm (Fig 1, S2 Fig in S1 File), a range determined through preliminary range-finding experiments to ensure reproducible lethality while allowing evaluation of intervention effects. This range avoided excessively high concentrations that would cause immediate death and lower concentrations that would require additional animal groups. Repeated trials confirmed stable exposure conditions within this concentration range. The inhaled dose was estimated (rather than directly measured) based on chamber concentration (ppm), exposure duration, and known respiratory parameters, including tidal volume and respiratory rate. This estimation approach is consistent with previously reported inhalation exposure models and provides a physiologically relevant approximation of internal exposure under acute conditions. The maximum observation time per experiment was 15 minutes, with immediate cessation upon any signs of apparent death or cessation of respiration and heartbeat. The survival time of each mouse was recorded. Although carbon monoxide (CO) may also be generated during combustion, the experimental conditions were optimized to prioritize cyanide-dominant exposure, and CO was not the primary variable of investigation in this study. Direct quantification of cyanide in blood was not feasible due to practical limitations in small animal models, including rapid onset of toxicity, limited blood sampling volume, and low detectable concentrations prior to death.

Therefore, an indirect assessment approach was applied. Specifically, the formation of cyanocobalamin following hydroxocobalamin administration was used as a functional indicator of cyanide exposure and detoxification. While this method does not provide a direct measurement of inhaled dose, it supports exposure estimation and evaluation of antidotal efficacy under the experimental conditions. After completing all group experiments, survival rates and median survival times were statistically analyzed. Surviving mice were monitored for at least one week post-experiment, with immediate medical intervention provided in case of distress.

The smoke chamber was designed to provide a controlled and reproducible toxic gas exposure model rather than to fully replicate the extreme physical conditions of real fire environments. During all experiments, ambient temperature and humidity were maintained within standard laboratory ranges to avoid confounding thermal injury, dehydration, or heat-induced physiological stress. This design allowed isolation of toxic gas–mediated effects while minimizing secondary injury mechanisms unrelated to chemical inhalation toxicity. Although real fire smoke contains a complex mixture of toxicants, cyanide and carbon monoxide are consistently identified as the primary contributors to early fire-related mortality. Therefore, this study focused on cyanide-dominant exposure to enable precise quantification of lethal concentrations and systematic evaluation of antidotal efficacy under defined conditions.

#### Drug formulation.

Two formulations of the combination drug were prepared:

1. **Low dose:** Hydroxocobalamin 25 mg + Deferoxamine 1 mg in one dose of 0.3M Tris buffer.
2. **High dose:** Hydroxocobalamin 150 mg + Deferoxamine 6 mg in one dose of 0.3M Tris buffer.

#### Animal welfare considerations.

Humane endpoints were predefined to minimize suffering, including unresponsiveness, loss of mobility to food or water, recurrent seizures, respiratory arrest, or hypothermia (<34 °C for >10 min). Animals meeting these criteria were euthanized within 5 minutes using isoflurane overdose followed by cervical dislocation. In the pilot study, 8 of 16 mice died before reaching humane criteria, informing lethal exposure settings. In the efficacy study, 4 control animals were euthanized and 8 (6 control, 2 treatment) died naturally, while 16 mice survived the 7-day observation period without reuse. Deaths were attributed to acute hypoxia or chemical asphyxiation (HCN/CO). All staff were trained and re-certified in welfare procedures, and the study complied with ARRIVE 2.0 and AVMA 2020 guidelines. Although lethal endpoints were scientifically necessary, every effort was made to minimize animal suffering.

#### Nebulization procedure.

Nebulization time for low-dose and high-dose combination drug administration in B6 mice. Mice were exposed to aerosolized Hydroxocobalamin and Deferoxamine under controlled conditions using a nebulizer. All mice were contained within a designated exposure unit to maintain consistent drug delivery conditions [9,14,18].

For the low-dose formulation (Hydroxocobalamin 25 mg + Deferoxamine 1 mg in 0.3M Tris buffer), the nebulization time averaged approximately 1 minute 35 seconds.

For the high-dose formulation (Hydroxocobalamin 150 mg + Deferoxamine 6 mg in 0.3M Tris buffer), the nebulization time averaged approximately 4 minutes 12 seconds (S1 Fig in S1 File).

#### Sample collection.

Blood and lung fluid samples were collected at specific time points post-administration (20 minutes, 1 hour, and 2 hours). Samples were analyzed to determine concentrations of hydroxocobalamin‌‌ and cyanocobalamin using UPLC-MS (Table 1) [11,12].

**Table 1:** Inhalation dynamics of differential dosages and concentrations in fire smoke models.

Sample	Dosage	Time	Hydroxocobalamin (ng/ml)	Cyanocobalamin (ng/ml)
**Blood**	Control	–	13.35 ± 3.35	8.90 ± 0.59
**Blood**	B12 25 mg + DFO 1 mg	20m	102.50 ± 7.42	10.71 ± 0.68
**Blood**	B12 25 mg + DFO 1 mg	1h	70.83 ± 18.65	10.79 ± 1.10
**Blood**	B12 25 mg + DFO 1 mg	2h	145.40 ± 71.22	10.60 ± 0.91
**Blood**	B12 150 mg + DFO 6 mg	20m	248.56 ± 41.22	16.58 ± 2.48
**Blood**	B12 150 mg + DFO 6 mg	1h	176.25 ± 16.26	13.10 ± 3.45
**Blood**	B12 150 mg + DFO 6 mg	2h	108.62 ± 14.36	6.87 ± 0.18
**Lung**	Control	–	—	—
**Lung**	B12 25 mg + DFO 1 mg	20m	598.77 ± 85.75	29.14 ± 5.44
**Lung**	B12 150 mg + DFO 6 mg	20m	904.81 ± 215.38	22.24 ± 2.71
**Lung**	B12 150 mg + DFO 6 mg	1h	338.56 ± 16.77	11.07 ± 1.07
**Lung**	B12 150 mg + DFO 6 mg	2h	234.90 ± 33.52	9.17 ± 1.64

Concentrations of Hydroxocobalamin and Cyanocobalamin in Blood and Lung Samples were quantified at predefined time points following nebulized administration, hydroxocobalamin concentrations were consistently higher in lung samples than in corresponding blood samples, indicating effective localized pulmonary delivery. The mice were divided into control and two experimental groups, receiving either 1X (25 mg/mL hydroxocobalamin + 1 mg/mL deferoxamine) or 5x (150 mg/mL hydroxocobalamin + 6 mg/mL deferoxamine), both reconstituted in 0.3M Tris buffer. Samples were collected at 20 minutes, 1 hour, and 2 hours post-administration. Data are expressed as mean ± standard deviation (ng/mL).

#### Sample analysis method.

In this study, we analyzed both blood and lung fluid samples using UPLC-MS (Ultra Performance Liquid Chromatography-Mass Spectrometry) for metabolomic profiling. The samples were collected, frozen, and stored at −20°C. For preparation, 60 µl of each sample was mixed with 240 µl of methanol to precipitate proteins. The mixture was vortexed, followed by centrifugation at 15,000g for 15 minutes at 4°C. Subsequently, 252 µl of the supernatant was transferred to an Eppendorf tube, vacuum-dried, and the residue reconstituted in 48 µl of water. The reconstituted solution was further centrifuged at 15,000g for 10 minutes at 4°C to remove precipitates. For the final analysis, 10 µl of the processed sample was injected into a Waters Xevo TQD mass spectrometer connected to a Waters ACQUITY UPLC system. Chromatographic separation was carried out on a BEH C18 column, and data was acquired in SRM (Selected Reaction Monitoring) mode with positive electrospray ionization (ESI+) [11,12].

#### Enhanced statistical analysis description for academic submission.

*Mann-Whitney U Test:*

Comparison: This test compared the survival times of two groups of mice, each consisting of 12 animals, under conditions of fire smoke exposure. The control group and the drug-treated group were assessed to determine if there was a significant difference in their overall survival times.

Statistical Difference: The Mann-Whitney U test yielded a p-value of 0.04, indicating a statistically significant difference in survival times between the control and drug groups, marked as significant (*p < 0.05, Table 2).

**Table 2:** Statistical analyses table.

Statistical Test	Comparison	P Value	Significance
**Log-Rank Test (Mantel–Cox)**	Survival distributions (control vs drug)	< 0.05	Significant
**Mann–Whitney U Test**	Median survival times (control vs drug)	0.04	Significant

*Log-Rank (Mantel-Cox) Test:*

Comparison: This test was utilized to compare the survival distributions over the 15-minute exposure period between the two groups. It specifically evaluated whether the probability of survival differed significantly over time between the control and drug-treated mice.

Statistical Difference: The results from the Log-Rank test would indicate whether the survival experiences of the two groups are statistically different, shedding light on the drug treatment’s efficacy.

## Results

Mouse exposure setup. A representative image shows a mouse positioned within a sealed exposure chamber where controlled HCN-rich smoke is introduced via the combustion of a small amount of melamine-based foam [5,9,10].

This exposure system enabled reproducible generation of HCN-dominant smoke under controlled laboratory conditions.

Survival Rate of Mice Exposed to Fire Smoke for 15 Minutes. This graph illustrates the survival percentage over time for two groups of mice (n = 10 per group), exposed to fire smoke. The control group (blue line) and drug-treated group (green line) were monitored for respiratory cessation or signs of evident mortality, at which point survival time was recorded. The x-axis represents survival time in minutes, capped at 15 minutes, and the y-axis depicts the survival percentage. The vertical dashed lines indicate the median survival times for each group. Control group (n = 10); drug-treated group (n = 10). Drug dose administered: Hydroxocobalamin 25 mg + Deferoxamine 1 mg in one dose of 0.3M Tris buffer.

Boxplot Comparing Survival Times of Control and Drug-Treated Mice. This boxplot displays the distribution of survival times for two groups of nine mice each, following a 15-minute exposure to fire smoke. Survival times were recorded at the moment of respiratory cessation or apparent death. The control group is represented in blue, and the drug-treated group in green. The boxplot shows the median, quartiles, and potential outliers in survival times for each group. Statistical analysis was performed using the Mann–Whitney U test (p = 0.04, Table 2).

Hydroxocobalamin and cyanocobalamin concentrations were further quantified in blood and lung fluid samples after nebulized administration, confirming pulmonary drug distribution and cyanocobalamin formation after smoke exposure (Table 1).

## Conclusion

In addition to these findings, our study highlights the importance of addressing cyanide exposure risks in fire scenarios. Tailored training and protective equipment for firefighters are essential for managing environments prone to high cyanide concentrations. Future investigations may further evaluate the feasibility of deploying nebulized antidotes in real-world fire scenarios, focusing on their potential to enhance firefighter safety and emergency medical interventions [9,10].

This study confirms the acute toxicity of cyanide in fire smoke and demonstrates the efficacy of hydroxocobalamin and deferoxamine in mitigating these dangers [4,5].

**Fig 2:**
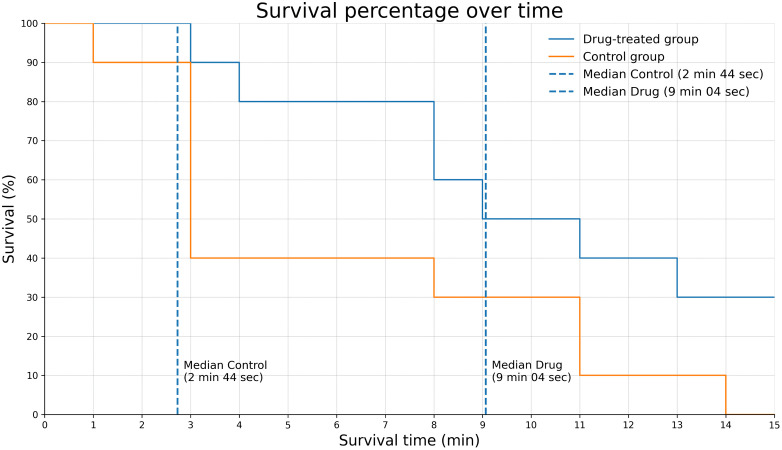
Survival rate plot legend.

**Fig 3:**
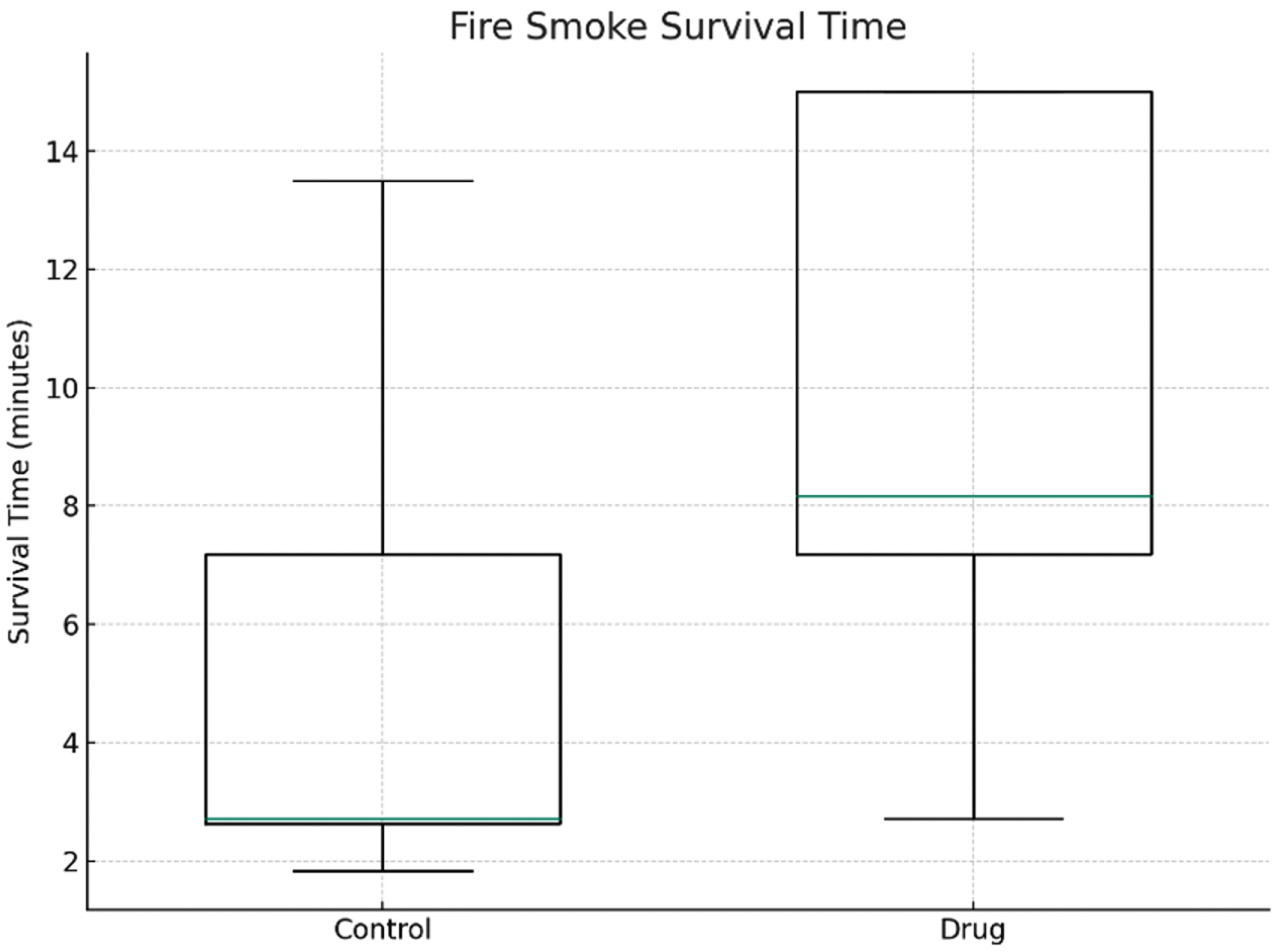
Boxplot legend.

Using controlled smoke chamber experiments, we observed significant differences in survival rates and times between the control group and the drug-treated group. Mice exposed to fire smoke without any protective intervention showed a 30% survival rate and a median survival time of 5 minutes, whereas those treated with hydroxocobalamin and deferoxamine achieved a 70% survival rate and a median survival time of 12 minutes (p < 0.05, Figs 2, 3, Tables 2, 3, S2 Fig in S1 File).

**Table 3:** Mouse survival analysis under varying HCN concentrations and exposure durations.

HCN (ppm)	Survival Time (Sec)	Mice number	Dead (Yes/NO)
**44**	540	M1	NO
**74**	336	M2	NO
**80**	453	M3	NO
**80**	450	M4	NO
**83**	372	M5	NO
**101**	345	M6	NO
**103**	453	M7	NO
**103**	387	M8	NO
**108**	279	M9	YES
**110**	258	M10	YES
**111**	270	M11	YES
**120**	315	M12	YES
**133**	228	M13	YES
**202**	180	M14	YES
**213**	180	M15	YES
**220**	147	M16	YES

Survival outcomes of mice were assessed under controlled exposure to different concentrations of hydrogen cyanide (HCN) and varied exposure durations [5]. Mice were placed in a controlled environment with specific HCN concentrations, and survival was monitored at intervals to determine the lethal dose and time-dependent effects of HCN exposure. The data are summarized in Table 3, where each row represents a distinct concentration-duration pairing, detailing the percentage of mice surviving at each condition. This analysis provides insights into the acute toxicity profile of HCN, establishing the correlation between concentration, exposure time, and survival probability.

The analysis of cyanide concentrations revealed consistent levels of 100–110 ppm in the smoke, providing a reliable baseline for assessing toxic exposure. Using advanced analytical methods, including UPLC-MS for drug concentration analysis and colorimetric methods for cyanide quantification, we evaluated exposure levels.

The aerosolized delivery method demonstrated efficient pulmonary delivery, with significantly higher drug concentrations detected in lung tissue compared to blood, indicating effective localized delivery (Table 4).

**Table 4:** Comparison of Hydroxocobalamin and cyanocobalamin concentrations in lung fluid (L) and smoke inhalation samples (S) across various experimental groups.

Drug Dose	Sample Type	Smoke Exposure	Hydroxocobalamin (ng/ml)	Cyanocobalamin (ng/ml)
**Low Dose**	Blood	No	47.43 ± 5.23	11.10 ± 1.14
**Low Dose**	Lung	No	60.37 ± 5.47	—
**Low Dose**	Blood	Smoke	119.43 ± 44.36	112.83 ± 23.26
**High Dose**	Blood	Smoke	135.67 ± 9.87	737.53 ± 16.96
**High Dose**	Lung	Smoke	1007.80 ± 698.50	650.30 ± 66.94
**High Dose**	Lung	Smoke	158.53 ± 9.94	266.10 ± 20.16

Mice received low dose, which included Hydroxocobalamin 25 mg and Deferoxamine 1 mg diluted in 0.3M Tris buffer, or high dose, which included Hydroxocobalamin 150 mg and Deferoxamine 6 mg diluted in 0.3M Tris buffer. Blood and lung fluid samples from smoke-exposed groups exhibited significantly higher Cyanocobalamin levels, supporting the conversion of Hydroxocobalamin to Cyanocobalamin in the presence of cyanide. Since each sample was processed independently, individual variability among mice may contribute to differences in drug concentrations. Additionally, the lung lavage process can result in variable drug recovery, which may lead to fluctuations in the measured concentrations. This table primarily serves to observe the impact of fire smoke on Hydroxocobalamin neutralization and its subsequent conversion to Cyanocobalamin.

Mean ± SEM values are presented for each condition (n = 3 per group). Statistical analysis was performed using a one-way ANOVA to compare Hydroxocobalamin and Cyanocobalamin concentrations across different groups, followed by a post-hoc Tukey’s test to identify significant pairwise differences. Significance was set at p < 0.05, with higher Cyanocobalamin levels observed in smoke-exposed groups compared to lung fluid samples.

The combination of hydroxocobalamin and deferoxamine offers a dual mechanism: hydroxocobalamin neutralizes cyanide by converting it into cyanocobalamin, while deferoxamine provides protection against oxidative damage caused by fire smoke (Table 1). This comprehensive approach underscores the potential of nebulized antidotes as a practical and effective solution for managing cyanide poisoning during fire incidents. Exposure to smoke induced significant inflammatory responses and tissue damage in lung tissue. Preventive drug administration demonstrated protective effects against smoke-induced lung injury, with dose-dependent improvements observed at higher drug doses (S6 Fig in S1 File). Inhalation of the tested drug, even at a high dosage, did not induce any detectable adverse hepatic changes. The histological findings suggest good hepatic safety and tolerability of the drug formulation (S7 Fig in S1 File).

While these findings provide experimental evidence for the efficacy of these antidotes, further research is needed to address limitations and explore long-term outcomes. This study lays a solid foundation for future clinical research on aerosolized hydroxocobalamin and deferoxamine, aiming to improve survival and safety in real fire scenarios. The insights gained here contribute to advancing fire smoke prevention strategies, shaping public safety policies, and enhancing emergency medical interventions to protect both civilians and firefighters.

## Discussion

Firefighters must be exceptionally prepared for the risk of cyanide poisoning in specific types of fires, including: (1) Residential Fires, (2) High-Rise Building Fires, (3) Industrial and Chemical Plant Fires, (4) Vehicle Fires, (5) Warehouse Fires, (6) Fires in Public Spaces with Synthetic Materials, (7) Transportation and Transit System Fires, and (8) Fires at Plastic Recycling or Disposal Facilities [4,15].

The results of our study highlight several key insights and implications for the management of cyanide toxicity in fire smoke and the efficacy of hydroxocobalamin and deferoxamine as antidotes. The potential role of aerosolized antidote delivery in real-world fire rescue operations has also been emphasized, particularly for rapid, non-invasive administration under hazardous conditions [14]. This discussion will explore the significance of our findings, address potential limitations, and propose directions for future research.

### Significance of findings

Our research has demonstrated that cyanide, a potent cellular toxin, poses significant risks during fire incidents, particularly in environments where nitrogen-containing materials are present. Recent advances in translational research have highlighted the need for novel and rapid therapeutic strategies to mitigate smoke inhalation injury, particularly targeting cyanide toxicity and oxidative stress [19]. The observed high lethality of cyanide at concentrations between 100–110 ppm in our smoke chamber experiments underscores the critical need for effective intervention strategies [16]. To establish a reliable model for generating high concentrations of hydrogen cyanide (HCN), we evaluated several combustion materials. Melamine-based foam was selected due to its ability to consistently generate high concentrations of hydrogen cyanide (HCN) under controlled combustion conditions. Given the high risk associated with these experiments, all combustion tests were conducted within a fume hood equipped with appropriate protective gear. Additionally, we informed and obtained permission from the Environmental and Safety Center at National Yang Ming Chiao Tung University prior to initiating the experiments.

#### Cyanide quantification.

To ensure reliability of cyanide exposure assessment, Hydrogen cyanide (HCN) concentrations were quantified using a colorimetric method based on reagent-induced color change.

The absorbance of this product was measured at a specific wavelength (λmax = 580 nm).

To accurately determine the cyanide concentration in our simulated fire smoke environment, we implemented a method using colorimetric data to establish a calibration curve for cyanide analysis. In addition, this quantification approach was used to compare with the HCN detector readings as a secondary verification method, ensuring consistency and reliability in cyanide measurement.

#### Calibration curve establishment.

Known concentrations of cyanide standard solutions (ranging from 0 to 110 ppm) were reacted with the reagent, and their absorbance values were measured at λmax = 580 nm (S5 Fig in S1 File).

Linear regression analysis was used to define the relationship between concentration and absorbance, establishing the relationship between concentration and absorbance.

#### Sample analysis.

Using the calibration curve, the measured absorbance value was converted to cyanide concentration.

#### Data processing.

Each sample was measured at least three times to reduce error, and the average value was used.

The cyanide concentrations calculated from the colorimetric data were used to assess cyanide exposure levels and were further combined with survival experiment data from mice to analyze the relationship between cyanide concentration and its lethality.

This strengthens confidence that the observed biological outcomes are directly associated with controlled cyanide exposure.

The significant improvement in survival rates and times in the drug-treated group compared to the control group provides strong evidence for the efficacy of hydroxocobalamin and deferoxamine in mitigating cyanide toxicity. Hydroxocobalamin’s ability to bind cyanide and convert it into the non-toxic cyanocobalamin was evident in the reduced blood cyanide levels in treated mice (S3, S4 Figs in S1 File). Deferoxamine’s role in protecting against free radical damage further supports the combined use of these drugs as a comprehensive treatment approach.

#### Recovery rate analysis.

To better understand the operational losses and ensure accurate drug quantification, we incorporated recovery rate analyses as supplemental data [11,12]. By using serum as the baseline and comparing recovery rates across formulations and nebulized samples, we identified potential degradation or deactivation of the drug. This approach allows us to validate the true concentrations of the nebulized drugs administered during the experiments, ensuring higher reliability in our efficacy assessments.

*Efficacy of Nebulized Drug Delivery*

The use of nebulization to deliver hydroxocobalamin and deferoxamine directly to the respiratory tract proved highly effective. The significantly higher concentrations of these drugs in lung tissue compared to blood suggest efficient local delivery, which is crucial for rapid detoxification and protection of the respiratory system. This method offers practical advantages for emergency response scenarios, where quick and targeted intervention is essential.

*Study Limitations*

Despite the promising results, our study has several limitations. That are inherent to the use of a controlled experimental model for toxic gas exposure. Firstly, the use of a controlled smoke chamber environment may not fully replicate the complexities and variabilities of real-world fire incidents. Factors such as varying smoke composition, environmental conditions, and the presence of other toxicants could influence the outcomes and should be considered in future studies.

Second, while mice serve as a relevant model for toxicological studies, there are physiological differences between mice and humans that may impact the generalizability of our findings. Further research involving other animal models or clinical trials would be necessary to validate the efficacy of these treatments in humans.

Third, the study focused on the immediate effects of cyanide and the antidotes. Long-term health effects and potential delayed toxicities were not included in the scope of this study. Extended observation periods and follow-up studies would be beneficial to understand any lasting impacts of cyanide exposure and treatment.

Fourth, although the inhaled dose was estimated using controlled exposure parameters, it was not directly measured. This limitation reflects practical constraints in small animal experiments, including rapid lethality and difficulty in obtaining reliable cyanide measurements prior to death.

To address this, cyanocobalamin formation was used as an indirect indicator of cyanide exposure and detoxification. While not a direct quantification method, it provides biologically relevant confirmation of exposure and antidote activity.

Importantly, the aim of this study was to evaluate protective efficacy under acute exposure conditions rather than to establish precise internal dose quantification.

While the sample size was sufficient to detect statistically significant differences in survival outcomes, further studies with larger cohorts may strengthen the robustness of these findings.

*Future Research Directions*

Building upon the present findings, future studies may explore:

Extended toxicity assessments to evaluate long-term outcomes following cyanide exposure and antidote administration. Alternative or combination antidote strategies to broaden protection against diverse toxicants present in fire smoke [17,18,20–22].

Field-based or simulation studies that more closely approximate real-world fire conditions [2,19]. Advanced detection technologies to enable rapid and accurate identification of cyanide and other toxic gases during emergency response [20,22]. Public health and safety policy development, including training protocols and antidote deployment guidelines for emergency responders [1].

## Supporting information

S1 File S1 Fig. Nebulization and animal grouping. The study utilized 8–10-week-old B6(C57BL/6) mice, assigned to receive either a low dose (25 mg/mL hydroxocobalamin + 1 mg/mL deferoxamine) or a high dose (150 mg/mL hydroxocobalamin + 6 mg/mL deferoxamine) via nebulization. Both formulations were reconstituted in 0.3 M Tris buffer. A minimum of three mice per group was included to ensure statistical reliability. Following inhalation, the mice were observed over an one-week period to assess therapeutic efficacy and toxicological safety. Throughout the observation period, none of the mice displayed adverse reactions, abnormal behavior, or mortality, demonstrating the tolerability of both dosing regimens. **S2 Fig. Mortality distribution in mice exposed to HCN.** A mortality distribution analysis was conducted to examine the relationship between hydrogen cyanide (HCN) concentration, exposure duration, and survival rate in mice. Figure 1 presents the mortality distribution across different HCN concentrations and exposure times, with each data point representing a unique concentration-duration combination. The distribution highlights the dose-dependent toxicity of HCN, where increased concentrations and prolonged exposure durations correlate with higher mortality rates. This distribution provides critical insights into the lethal thresholds of HCN, helping to establish safe exposure limits and improve understanding of acute toxicity mechanisms. **S3 Fig. Calibration curve of hydroxocobalamin.** The compound analyzed was hydroxocobalamin, with a correlation coefficient of r = 0.998196 and r^2^ = 0.996395. The calibration curve equation was 14.3037 * x + −15.7776, using an external standard response type based on area. The curve type was linear, with the origin included, and the weighting was 1/x, with no axis transformation applied. **S4 Fig. Calibration curve of cyanocobalamin.** The compound analyzed was cyanocobalamin, with a correlation coefficient of r = 0.996805 and r^2^ = 0.993620. The calibration curve equation was 30.8594 * x + −9.71672, using an external standard response type based on area. The curve type was linear, with the origin included, and the weighting was 1/x, with no axis transformation applied. **S5 Fig. Calibration curve for hydrogen cyanide (HCN) concentration determination.** The curve was obtained through quadratic regression analysis of measured HCN concentrations (ppm vs. mg/L) using colorimetric assays. The fitted equation is y = 0.00022x^2^ − 0.0536x + 3.2820, with a high correlation coefficient (R^2^) of 0.9989, indicating excellent fit quality and reliability for quantitative analyses. **S6 Fig. Lung histopathological changes after smoke inhalation and preventive drug administration.** Representative hematoxylin and eosin (H&E) stained lung tissue sections under 4x (upper panel, scale bar: 1000 μm) and 20x magnification (lower panel, scale bar: 200 μm). Higher-dose treatment groups demonstrated increased pulmonary drug concentrations and improved survival outcomes compared with lower-dose groups. Preventive drug administration demonstrated protective effects against smoke-induced lung injury, with improved histological outcomes observed at higher drug doses. **S7 Fig. Hepatic histopathological evaluation after drug inhalation at high dosage.** Representative liver sections stained with hematoxylin and eosin (H&E) under 4x (upper panel, scale bar: 1000 μm) and 20x magnification (lower panel, scale bar: 200 μm). Inhalation of the tested drug formulation, even at the high-dose level, did not induce any detectable adverse hepatic changes. Histopathological examination revealed no observable pathological alterations in liver tissue following drug administration. **S1 Table. Baseline serum recalculated relative rate.** This table shows the recalculated relative recovery values of the drug using serum as the baseline. The data may reflect potential losses during the operational process, including sample preparation loss, matrix effects, dilution correction, or possible drug degradation. **S2 Table. Recalculated relative recovery for 25 mg/mL hydroxocobalamin + 1 mg/mL deferoxamine.** This table demonstrates the recalculated relative recovery values observed when using the low-dose formulation. It provides insights into the relative measured concentrations and potential operational losses of the nebulized drug under the experimental conditions. Blood: Refers to samples collected from blood serum. Lung: Refers to samples collected from lung tissue or lung fluid. **S3 Table. Recalculated relative recovery for 150 mg/mL hydroxocobalamin.** This table displays the recalculated relative recovery values for the high-dose formulation. By comparing this with the baseline serum recalculated relative recovery, one can infer the relative concentrations measured after nebulization and the operational losses during the process. Blood: Refers to samples collected from blood serum. Lung: Refers to samples collected from lung tissue or lung fluid. SL: Refers to lung fluid samples collected from lung tissues after smoke exposure.
(ZIP)
